# Long-term survival following upgrade compared with *de novo* cardiac resynchronization therapy implantation: a single-centre, high-volume experience

**DOI:** 10.1093/europace/euab059

**Published:** 2021-05-25

**Authors:** Walter Richard Schwertner, Anett Behon, Eperke Dóra Merkel, Márton Tokodi, Attila Kovács, Endre Zima, István Osztheimer, Levente Molnár, Ákos Király, Roland Papp, László Gellér, Luca Kuthi, Boglárka Veres, Annamária Kosztin, Béla Merkely

**Affiliations:** Heart and Vascular Centre, Semmelweis University, Városmajor 68, H-1122 Budapest, Hungary

**Keywords:** Cardiac resynchronization therapy, *De novo* CRT, Upgrade, All-cause mortality, Complication

## Abstract

**Aims:**

Patients with a pacemaker or implantable cardioverter-defibrillator are often considered for cardiac resynchronization therapy (CRT). However, limited comprehensive data are available regarding their long-term outcomes.

**Methods and results:**

Our retrospective registry included 2524 patients [1977 (78%) *de novo*, 547 (22%) upgrade patients] with mild to severe symptoms, left ventricular ejection fraction ≤35%, and QRS ≥ 130ms. The primary outcome was the composite of all-cause mortality, heart transplantation (HTX), or left ventricular assist device (LVAD) implantation; secondary endpoints were death from any cause and post-procedural complications. In our cohort, upgrade patients were older [71 (65–77) vs. 67 (59–73) years; *P* < 0.001], were less frequently females (20% vs. 27%; *P* = 0.002) and had more comorbidities than *de novo* patients. During the median follow-up time of 3.7 years, 1091 (55%) *de novo* and 342 (63%) upgrade patients reached the primary endpoint. In univariable analysis, upgrade patients exhibited a higher risk of mortality/HTX/LVAD than the *de novo* group [hazard ratio (HR): 1.41; 95% confidence interval (CI): 1.23–1.61; *P* < 0.001]. However, this difference disappeared after adjusting for covariates (adjusted HR: 1.12; 95% CI: 0.86–1.48; *P* = 0.402), or propensity score matching (propensity score-matched HR: 1.10; 95% CI: 0.95–1.29; *P* = 0.215). From device-related complications, lead dysfunction (3.1% vs. 1%; *P* < 0.001) and pocket infections (3.7% vs. 1.8%; *P* = 0.014) were more frequent in the upgrade group compared to *de novo* patients.

**Conclusion:**

In our retrospective analysis, upgrade patients had a higher risk of all-cause mortality than *de novo* patients, which might be attributable to their more significant comorbidity burden. The occurrence of lead dysfunction and pocket infections was more frequent in the upgrade group.

**What’s new?**

- In a patient cohort including >2500 CRT recipients with long-term follow-up data, we demonstrated a 41% higher risk of mortality/HTX/LVAD implantation in upgrade compared to *de novo* patients. This observed difference in outcomes might be attributable to their more significant comorbidity burden as it disappeared after adjusting for relevant clinical covariates or performing propensity score matching.
- Cardiac resynchronization therapy upgrade procedures were associated with higher rates of lead dysfunction or fracture and pocket infection than *de novo* CRT implantation.

## Introduction

Cardiac resynchronization therapy (CRT) is an effective treatment in symptomatic heart failure (HF) patients with reduced left ventricular ejection fraction (LVEF) and left bundle branch block (LBBB).^1^^,^^2^ Several prospective randomized trials have shown that in this patient population, resynchronization therapy is associated with improved symptoms, quality of life, left ventricular function,^1^ and reduced cardiovascular mortality.^2^ However, there are patient subgroups whose long-term outcomes are less predictable; thus, further investigations or more complex prediction models are required.^3^

Most randomized clinical trials excluded patients with prior conventional pacemakers (PM) or implantable cardioverter-defibrillators (ICD). Moreover, no randomized trials were conducted regarding the effects of CRT upgrade in patients with chronic right ventricular pacing (RVP). So far, the ongoing BUDAPEST-CRT Upgrade Study (NCT02270840) is the first prospective randomized controlled trial that will address this question.^4^

Chronic RVP can induce mechanical dyssynchrony similarly to the LBBB, leading to a reduction in ventricular function.^5^ Moreover, the high percentage of RVP is associated with an increased risk of HF and atrial fibrillation (AF) events.^6^ Nonetheless, these unfavourable events might be prevented by performing a CRT upgrade procedure.^5^

Despite the increasing number of CRT upgrade procedures,^7^ scientific evidence is controversial concerning the long-term outcomes of upgrade patients. Thus, in our retrospective cohort study, we aimed to compare the long-term survival and complications rate of patients after *de novo* or upgrade CRT procedures.

## Methods

### Patient population

Our registry comprised patients (*n* = 2524) who underwent CRT upgrade or *de novo* implantation between 28 July 2000 and 6 September 2018 at the Heart and Vascular Centre of Semmelweis University. Patients were considered for CRT as per the current guidelines: New York Heart Association (NYHA) functional Class II to IVa, LVEF ≤35%, and QRS width ≥130 ms, despite the optimal medical therapy.^7^ Patients with no active health insurance at the time of implantation were excluded.

### Baseline evaluation

Data were extracted retrospectively from paper-based and electronic medical records. Anthropometric-, laboratory, echocardiographic-, and ECG parameters, NYHA functional class, and comorbidities were collected for each patient at baseline. Echocardiographic measurements were performed 7 (0–29) days, whereas devices (of the upgrade patients) were interrogated 10 (0–40) days before the procedures.

The examination complies with the Declaration of Helsinki, and it was approved by the Regional and Institutional Committe of Science and Research Ethics (Approval No. 161-0/2019).

### Endpoints

The primary composite endpoint was defined as mortality from any cause, implantation of left ventricular assist device (LVAD), or heart transplantation (HTX). Follow-up data [status (dead or alive), date of death] were obtained for all patients by querying the National Health Insurance Database of Hungary in September 2019. Secondary endpoints were death from any cause and peri- and post-procedural complications.

### Cardiac resynchronization therapy implantation

Left ventricular lead implantations were performed via cephalic or subclavian vein access using a transvenous approach to a coronary sinus side branch. In unsuccessful cases, transseptal lead implantation or epicardial implantation from mini-thoracotomy were performed. During the transvenous process, to assess the optimal side branch, a venogram balloon catheter was used, and images were recorded. Left ventricular leads were positioned, preferably to a lateral or postero-lateral coronary sinus side branch avoiding the apex, whereas right ventricular leads were positioned to a septal position.

### Statistical analysis

Statistical analysis was performed using SPSS (version 25.0, IBM, Armonk, NY, USA), GraphPad Prism (version 8, Inc., GraphPad Software, San Diego, CA, USA), and RStudio (version 1.8, RStudio PBC, Boston, MA, USA). To determine whether the data are distributed normally, we performed Shapiro–Wilk tests. In case of normal distribution, continuous variables were expressed as mean ± standard deviation (SD), whereas not normally distributed parameters were expressed as median and inter-quartile range (IQR). To compare continuous variables within the same group, paired Student’s *t*-test or paired Wilcoxon rank test was performed, as appropriate. Depending on normality, continuous variables between groups were compared using unpaired Student’s *t*-test or Mann–Whitney *U* test. To compare categorical variables, *χ*^2^ or Fisher’s exact tests were performed. Time-to-event analyses were performed using log-rank tests, univariable and multivariable Cox regression analyses. We performed propensity score matching in R (version 3.6.3, R Foundation for Statistical Computing, Vienna, Austria) using the MatchIt package (version 3.0.2). After replacing the missing values with the mean of the non-missing cases, propensity score matching was performed using the nearest neighbour matching (distance calculated with the logistic regression method). To assess the impact of the implantation date on the primary composite endpoint, we created dummy variables based on the year of CRT implantation. A *P*-value of < 0.05 was considered statistically significant.

## Results

### Patient characteristics

A total of 2524 patients were included in our registry, of whom 1977 (78%) received CRT as a primary device, and 547 (22%) underwent an upgrade procedure. In the total cohort, the median follow-up time was 3.7 (1.9–6.4) years.

Among the upgrade CRT patients, 142 (26%) had VVI and 119 (22%) VVI-ICD, 164 (30%) had DDD and 74 (14%) DDD-ICD, 32 (6%) had VDD, and 10 (2%) VDD-ICD devices prior to CRT implantation. In those with previous devices, the median duration of RVP was 4.5 (2.1–8.1) years, whereas the median RVP rate was 95 (62–99) % before the upgrade CRT procedure.

Regarding the baseline clinical characteristics, upgrade patients were significantly older [71 (65–77) vs. 67 (59–73) years; *P* < 0.001], were more likely to have ischaemic aetiology [328 (60%) vs. 908 (46%); *P* < 0.001] accompanied with a higher prevalence of prior myocardial infarction [262 (48%) vs. 712 (36%); *P* < 0.001] and coronary artery bypass graft procedures [105 (19%) vs. 228 (12%); *P* < 0.001] (*Table 1*). While, NYHA III/IV functional status [265 (52%) vs. 916 (59%); *P* = 0.007] and female sex [110 (20%) vs. 527 (27%); *P* = 0.002] were less common in the upgrade patient group, they suffered more frequently from AF [258 (47%) vs. 692 (35%); *P* < 0.001] and ventricular arrhythmias [181 (33%) vs. 421 (22%); *P* < 0.001]. Moreover, chronic kidney disease (CKD) [268 (49%) vs. 668 (34%); *P* < 0.001] was more common based on estimated glomerular filtration rate (eGFR) [52.8 (39.7–62.8) mL/min/1.73 m^2^ vs. 63 (46.6–78.4) mL/min/1.73 m^2^; *P* < 0.001] and creatinine levels [111 (89–142) vs. 98 (79–126) µmol/L; *P* < 0.001] in the upgrade group compared to the *de novo* patients (*Table 1*). Among patients with a previous device, paced QRS duration was significantly broader compared with the *de novo* group (174.1 ± 30.6 vs. 158.3 ± 26.0 ms; *P* < 0.001).

**Table 1:** Baseline clinical characteristics of *de novo* and upgrade CRT patients

	All patients (*n* = 2524)	*De novo* CRT (*n* = 1977)	Upgrade CRT (*n* = 547)	*P*-value
Age (years), median (IQR)	68 (61–74)	67 (59–73)	71 (65–77)	<0.001
Sex (female), *n* (%)	637 (25)	527 (27)	110 (20)	0.002
NYHA III/IV, *n* (%)	1181 (57)	916 (59)	265 (52)	0.007
BMI (kg/m^2^), median (IQR)	27.4 (24.6–30.8)	27.4 (24.5–30.7)	27.7 (24.7–30.9)	0.383
QRS width (ms), mean ± SD	161.6 ± 27.8	158.3 ± 26.0	174.1 ± 30.6	<0.001
Ischaemic aetiology, *n* (%)	1236 (49)	908 (46)	328 (60)	<0.001
Medical history				
MI, *n* (%)	974 (39)	712 (36)	262 (48)	<0.001
PCI, *n* (%)	739 (29)	560 (28)	179 (33)	0.056
CABG, *n* (%)	333 (13)	228 (12)	105 (19)	<0.001
HT, *n* (%)	1819 (72)	1416 (72)	403 (74)	0.360
DM, *n* (%)	927 (37)	724 (37)	203 (37)	0.841
Type II DM, *n* (%)	750 (29)	598 (30)	152 (28)	0.290
Atrial fibrillation, *n* (%)	950 (38)	692 (35)	258 (47)	<0.001
Ventricular arrhythmia, *n* (%)	602 (24)	421 (22)	181 (33)	<0.001
CRT-D implantation, *n* (%)	1366 (54)	1051 (53)	315 (58)	0.066
Laboratory parameters				
NT-proBNP (pg/mL), median (IQR)	2757 (1588–3756)	2717 (1424–3139)	2873 (1640–4644)	0.054
Creatinine (µmol/L), median (IQR)	101 (81–131)	98 (79–126)	111 (89–142)	<0.001
eGFR (mL/min/1.73 m^2^), median (IQR)	60 (44.9–76.2)	63 (46.6–78.4)	52.8 (39.7–62.8)	<0.001
CKD, *n* (%)	936 (37)	668 (34)	268 (49)	<0.001
Echocardiographic parameters				
LVEF (%), median (IQR)	28 (24–33)	28 (24–33)	29 (25–35)	0.014
LVEDd (mm), mean ± SD	63.8 ± 9.5	64.1 ± 9.5	63.0 ± 9.4	0.037
LVESd (mm), mean ± SD	53.6 ± 10.3	54.1 ± 10.2	52.3 ± 10.6	0.007
Medical treatment				
Loop diuretics, *n* (%)	1829 (80)	1413 (79)	416 (82)	0.214
Thiazide diuretics, *n* (%)	548 (24)	416 (23)	132 (26)	0.239
β-blockers, *n* (%)	2043 (89)	1584 (89)	459 (90)	0.424
MRA, *n* (%)	1557 (68)	1203 (67)	354 (69)	0.390
ACE-I/ARB, *n* (%)	2111 (92)	1656 (92)	455 (89)	0.022
Amiodarone, *n* (%)	619 (27)	447 (25)	172 (34)	0.001
OAC, *n* (%)	772 (34)	538 (30)	234 (46)	<0.001

Continuous variables were listed as mean ± SD or median/IQR, and categorical variables were listed as *n* (%). Continuous variables were compared using unpaired Student’s *t*-test or Mann–Whitney *U* test, while categorical variables were compared using *χ*^2^ or Fisher’s exact tests. *P*-values refer to differences between the *de novo* and the upgrade CRT groups.

ACE-I, angiotensin-converting enzyme inhibitors; ARB, angiotensin receptor blocker, BMI, body mass index; CABG, coronary artery bypass grafting; CKD, chronic kidney disease; CRT-D, cardiac resynchronization therapy defibrillator; DM, diabetes mellitus; eGFR, estimated glomerular filtration rate; HT, hypertension; IQR, inter-quartile range; LVEDd, left ventricular end-diastolic diameter; LVEF, left ventricular ejection fraction; LVESd, left ventricular end-systolic diameter; MI, myocardial infarction; MRA, mineralocorticoid receptor antagonists; NT-proBNP, N-Terminal pro-B-Type Natriuretic Peptide; NYHA, New York Heart Association class; OAC, oral anticoagulant; PCI, percutaneous coronary intervention; SD, standard deviation.

Due to the higher rate of AF in the upgrade patients, they received oral anticoagulation (OAC) [234 (46%) vs. 538 (30%); *P* < 0.001] and amiodarone [172 (34%) vs. 447 (25%); *P* = 0.001] more frequently. However, the prescription rate of angiotensin-converting enzyme inhibitors (ACE-I) or angiotensin II receptor blockers (ARB) [455 (89%) vs. 1656 (92%); *P* = 0.022] was lower among upgrade patients (*Table 1*).

Regarding echocardiographic parameters, the upgrade group had higher baseline LVEF [29 (25–35)% vs. 28 (24–33)%; *P* = 0.014] and smaller left ventricular diameters (LVEDd 63.0 ± 9.4 vs. 64.1 ± 9.5 mm; *P* = 0.037, LVESd 52.3 ± 10.6 vs. 54.1 ± 10.2 mm; *P* = 0.007) compared with the *de novo* group (*Table 1*).

### Long-term outcomes—primary endpoint

During the median follow-up time of 3.7 (1.9–6.4) years, 1433 (56.8%) patients reached the composite primary endpoint [1091 (55.2%) patients in the *de novo* and 342 (62.5%) in the upgrade CRT group]. Overall, 1057 (53.5%) *de novo* and 334 (61.1%) upgrade patients died, 31 (1.6%) *de novo* and 8 (1.5%) upgrade patients had HTX and 3 (0.2%) *de novo* patients underwent LVAD implantation.

**Figure 1:**
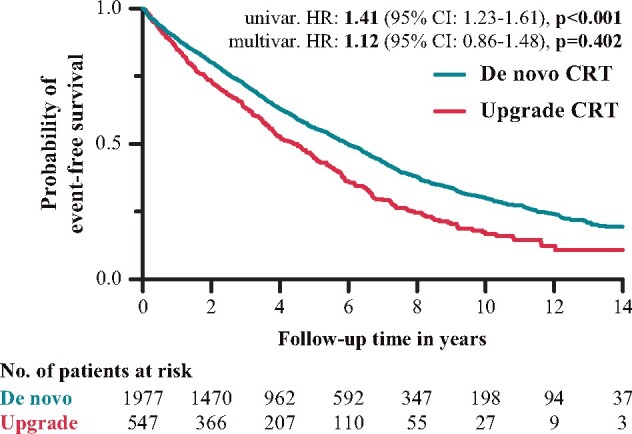
Kaplan–Meier curves for the primary composite endpoint in *de novo* and upgrade patients. CI, confidence interval; CRT, cardiac resynchronization therapy; HR, hazard ratio.

The univariable Cox regression analysis showed more unfavourable primary composite outcomes in the upgrade group [hazard ratio (HR): 1.41; 95% confidence interval (CI): 1.23–1.61; *P* < 0.001] compared with *de novo* CRT patients (*Figure 1*). However, when multivariable Cox regression analysis was performed, including the relevant clinical covariates (ACE-I/ARB, age, amiodarone, AF, intrinsic/paced QRS duration time, ischaemic aetiology, LVEDd, LVESd, LVEF, NYHA Class III/IV, OAC, serum creatinine, sex, and ventricular arrhythmia), the previously described difference could not be observed (HR: 1.12; 95% CI: 0.86–1.48; *P* = 0.402) (*Figure 1*). In our patient cohort, AF (HR: 1.31; 95% CI: 1.02–1.69; *P* = 0.032), female sex (HR: 0.72; 95% CI: 0.54–0.96; *P* = 0.025), ischaemic HF aetiology (HR: 1.66; 95% CI: 1.32–2.09; *P* < 0.001), NYHA class III/IV (HR: 1.38; 95% CI: 1.09–1.75; *P* = 0.009), and serum creatinine (HR: 1.01; 95% CI: 1.00–1.01; *P* < 0.001) were found to be independent predictors of the primary composite endpoint (Supplementary material online, *Table S1*).

**Figure 2:**
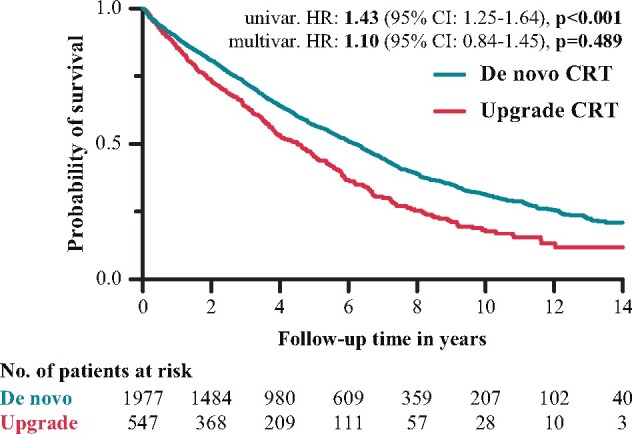
(*A*) Kaplan–Meier curves for the primary composite endpoint in the propensity score-matched *de novo* and upgrade CRT groups. (*B*) Kaplan–Meier curves of all-cause death in the propensity score-matched *de novo* and upgrade CRT group. CI, confidence interval; CRT, cardiac resynchronization therapy; HR, hazard ratio.

In addition, propensity score matching was performed to compare the outcomes of the two groups after eliminating the differences in the relevant clinical covariates. In this analysis, each patient in the upgrade group (*n* = 547) was matched with a patient from the *de novo* group who was very similar across the following covariates: age, AF, eGFR, HF aetiology, LVEF, NYHA functional class, sex, QRS duration, and ventricular arrhythmia (Supplementary material online, *Table S2*). When we compared the matched *de novo* and upgrade groups, we found no significant difference in the risk of reaching the composite primary endpoint (propensity score-matched HR: 1.10; 95% CI: 0.95–1.29; *P* = 0.215) (*Figure 2A*).

To determine whether the date of implantation affects the primary composite endpoint, we created dummy coded variables based on the year of the CRT implantation dates and a dichotomous variable using the year 2013 as the cut-off. However, neither the dummies nor the dichotomized (adjusted HR: 0.88; 95% CI: 0.60–1.30; *P* = 0.532) variables were found to be predictors of the composite endpoint.

### Long-term outcomes—all-cause mortality

During the median follow-up time of 3.8 (1.9–6.5) years, 1409 (55.8%) patients died, 1071 (54.2%) in the *de novo*, and 338 (61.7%) in the upgrade CRT group.

**Figure 3:**
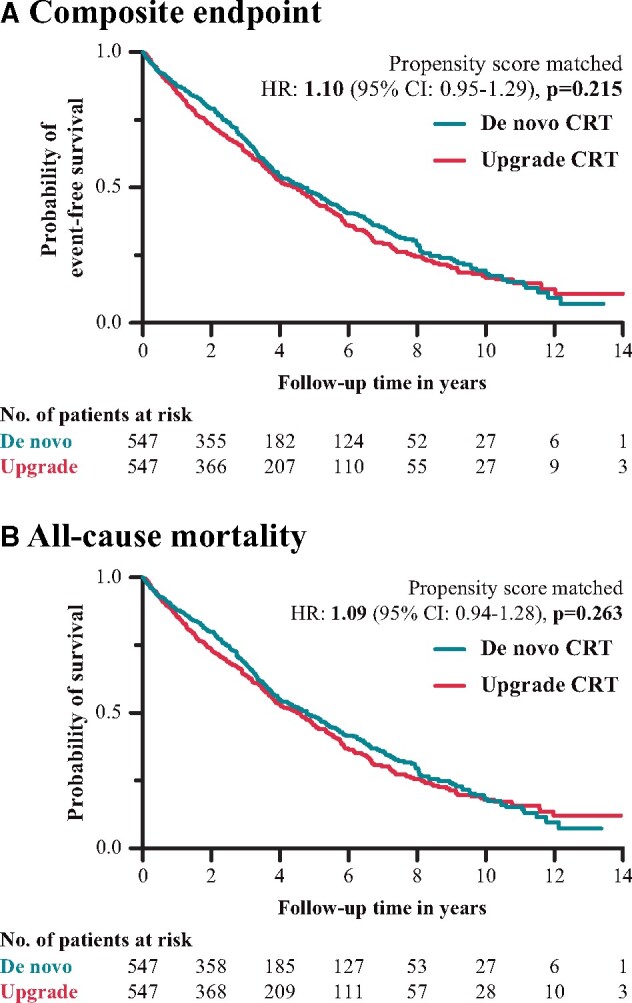
Kaplan–Meier curves of all-cause death in *de novo* and upgrade patients. CI, confidence interval; CRT, cardiac resynchronization therapy; HR, hazard ratio.

The univariable Cox regression analysis showed a 43% higher rate of all-cause mortality in the upgrade CRT group compared with *de novo* patients (HR: 1.43; 95% CI: 1.25–1.64; *P* < 0.001) (*Figure 3*). After adjusting for the relevant clinical covariates (ACE-I/ARB, age, amiodarone, AF, intrinsic/paced QRS duration time, ischaemic aetiology, LVEDd, LVESd, LVEF, NYHA class III/IV, OAC, serum creatinine, sex, and ventricular arrhythmia), multivariable Cox regression analysis showed a similar risk of all-cause mortality in the two patient groups (HR: 1.10; 95% CI: 0.84–1.45; *P* = 0.489) (*Figure 3*). In our patient cohort, age (HR: 1.02; 95% CI: 1.01–1.03; *P* = 0.002), AF (HR: 1.41; 95% CI: 1.10–1.81; *P* = 0.008), female sex (HR: 0.74; 95% CI: 0.56–0.99; *P* = 0.042), ischaemic HF aetiology (HR: 1.59; 95% CI: 1.26–2.00; *P* < 0.001), LVESd (HR: 1.04; 95% CI: 1.00–1.08; *P* = 0.039), NYHA Class III/IV (HR: 1.35; 95% CI: 1.06–1.71; *P* = 0.015), and serum creatinine (HR: 1.01; 95% CI: 1.00–1.01; *P* < 0.001) were found to be independent predictors of all-cause mortality (Supplementary material online, *Table S3*). Comparing the propensity-matched cohorts, no significant difference was found between *de novo* and upgrade groups in all-cause mortality (propensity score-matched HR: 1.09; 95% CI: 0.94–1.28; *P* = 0.263) (*Figure 2B*).

### Subgroup analysis

**Figure 4:**
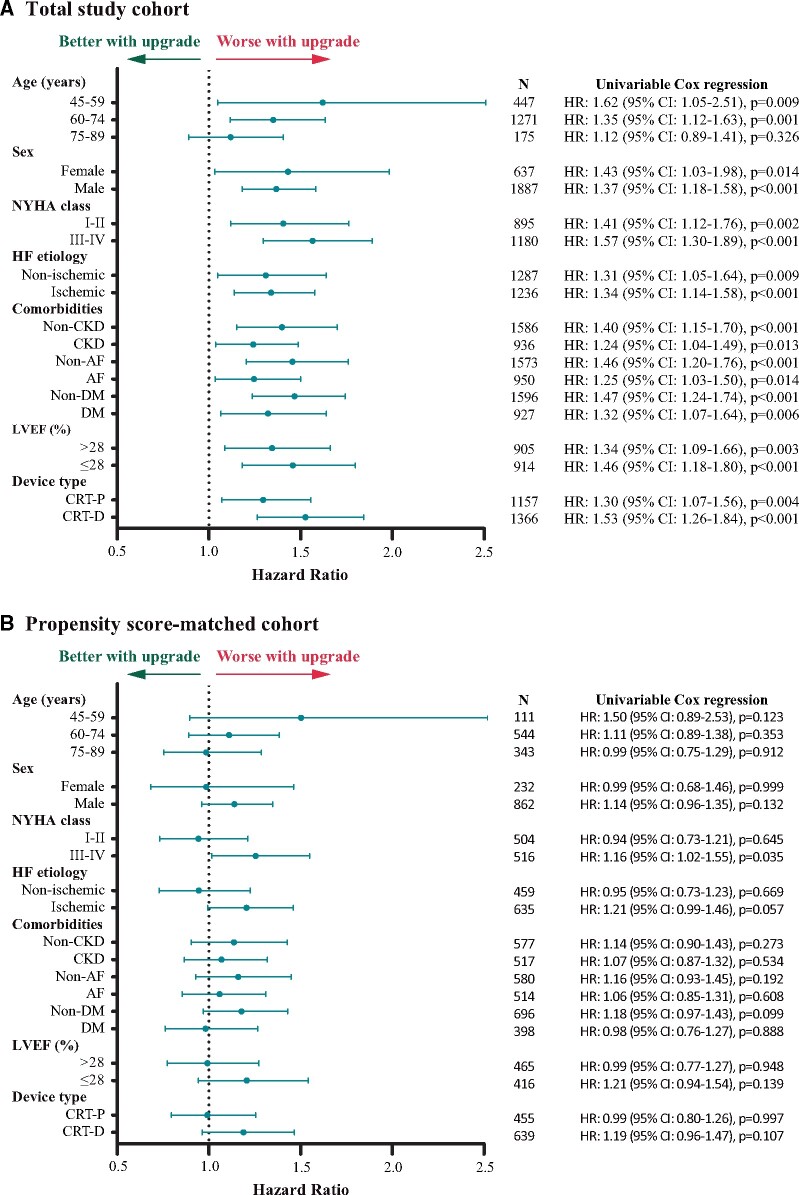
(*A*) Forest plot of subgroups based on the composite endpoint of the total cohort. (*B*) Forest plot of subgroups based on the composite endpoint of the propensity score-matched cohort. AF, atrial fibrillation; CI, confidence interval; CKD, chronic kidney disease; CRT, cardiac resynchronization therapy; CRT-D, cardiac resynchronization therapy-defibrillator; CRT-P, cardiac resynchronization therapy pacemaker; DM, diabetes mellitus; HR, hazard ratio; LVEF, left ventricular ejection fraction; NYHA, New York Heart Association.

In the subgroups created based on age at CRT implantation, sex, NYHA functional class, heart failure aetiology, comorbidities, ejection fraction, and CRT type, additional analyses were performed to assess differences in the composite endpoint between *de novo* and upgrade patients. In the total cohort, upgrade patients showed a higher risk of the composite endpoint in all subgroups, except for the 75–89 age group, where comparable outcomes were found between the *de novo* and upgrade patients (HR: 1.12; 95% CI: 0.89–1.41; *P* = 0.326) (*Figure 4A*). When the propensity score-matched cohort was analysed, we observed a similar risk of the composite endpoint in all subgroups, except for patients with severe symptoms (NYHA III–IV), where upgrade patients exhibited a higher risk compared with *de novo* patients (HR: 1.26; 95% CI: 1.02–1.55; *P* = 0.035) (*Figure 4B*).

### Complications

In the total cohort, the most frequent complications were lead displacement (6.5%) and phrenic nerve stimulation (3%). Upgrade patients suffered more often from lead dysfunction or fracture [17 (3.1%) vs. 19 (1.0%); *P* < 0.001], pocket infection [20 (3.7%) vs. 36 (1.8%); *P* = 0.014] than patients who underwent *de novo* CRT procedures (*Table 2*). The less frequent complications were coronary sinus dissection (0.9%), pericardial tamponade (0.4%), infective endocarditis (0.4%), and haemothorax (0.2%), in which no significant difference could be observed between the patient groups.

**Table 2:** Complications associated with *de novo* or upgrade CRT implantation procedures (with and without performing propensity score matching)

	All patients (*n* = 2524)	*De novo* CRT (*n* = 1977)	Upgrade CRT (*n* = 547)	*P*-value	PSM all patients (*n* = 1094)	PSM *de novo* CRT (*n* = 547)	PSM upgrade CRT (*n* = 547)	*P*-value
Pneumothorax, *n* (%)	30 (1.2)	28 (1.4)	2 (0.4)	0.045	10 (0.9)	8 (1.5)	2 (0.4)	0.108
1 month	30 (1.2)	28 (1.4)	2 (0.4)	0.045	10 (0.9)	8 (1.5)	2 (0.4)	0.108
1–12 months	–	–	–	–	–	–	–	–
After 12 months	–	–	–	–	–	–	–	–
Coronary sinus dissection, *n* (%)	22 (0.9)	15 (0.8)	7 (1.3)	0.295	11 (1)	4 (0.7)	7 (1.3)	0.547
1 month	22 (0.9)	15 (0.8)	7 (1.3)	0.295	11 (1)	4 (0.7)	7 (1.3)	0.547
1–12 months	–	–	–	–	–	–	–	–
After 12 months	–	–	–	–	–	–	–	–
Pericardial tamponade, *n* (%)	9 (0.4)	7 (0.4)	2 (0.4)	0.999	4 (0.4)	2 (0.4)	2 (0.4)	>0.999
1 month	6 (0.2)	4 (0.2)	2 (0.4)	0.616	3 (0.3)	1 (0.2)	2 (0.4)	>0.999
1–12 months	3 (0.1)	3 (0.2)	0 (0.0)	>0.999	1 (0.1)	1 (0.2)	0 (0.0)	>0.999
After 12 months	–	–	–	–	–	–	–	
Lead displacement, *n* (%)	163 (6.5)	130 (6.6)	33 (6.0)	0.695	71 (6.5)	38 (6.9)	33 (6.0)	0.624
1 month	61 (2.4)	52 (2.6)	9 (1.6)	0.210	26 (2.4)	17 (3.1)	9 (1.6)	0.164
1–12 months	56 (2.2)	44 (2.2)	12 (2.2)	>0.999	27 (2.5)	15 (2.7)	12 (2.2)	0.698
After 12 months	45 (1.8)	33 (1.7)	12 (2.2)	0.464	18 (1.6)	6 (1.1)	12 (2.2)	0.234
Lead dysfunction/fracture, *n* (%)	36 (1.4)	19 (1.0)	17 (3.1)	<0.001	21 (1.9)	4 (0.7)	17 (3.1)	0.007
1 month	4 (0.2)	3 (0.2)	1 (0.2)	>0.999	2 (0.2)	1 (0.2)	1 (0.2)	>0.999
1–12 months	8 (0.3)	3 (0.2)	5 (0.9)	0.015	6 (0.5)	1 (0.2)	5 (0.9)	0.218
After 12 months	24 (1.0)	13 (0.7)	11 (2.0)	0.010	13 (1.2)	2 (0.4)	11 (2.0)	0.022
Phrenic nerve stimulation, *n* (%)	75 (3.0)	58 (2.9)	17 (3.1)	0.778	32 (2.9)	15 (2.7)	17 (3.1)	0.858
1 month	53 (2.1)	45 (2.3)	8 (1.5)	0.312	19 (1.7)	11 (2.0)	8 (1.5)	0.645
1–12 months	12 (0.5)	6 (0.3)	6 (1.1)	0.028	9 (0.8)	3 (0.5)	6 (1.1)	0.506
After 12 months	10 (0.4)	7 (0.3)	3 (0.5)	0.460	4 (0.4)	1 (0.2)	3 (0.5)	0.624
Bleeding/pocket haematoma, *n* (%)	28 (1.1)	16 (0.8)	12 (2.2)	0.010	18 (1.6)	6 (1.1)	12 (2.2)	0.234
1 month	24 (1.0)	14 (0.7)	10 (1.8)	0.024	16 (1.5)	6 (1.1)	10 (1.8)	0.451
1–12 months	1 (0.04)	0 (0.0)	1 (0.2)	0.217	1 (0.1)	0 (0.0)	1 (0.2)	>0.999
After 12 months	3 (0.1)	2 (0.1)	1 (0.2)	0.520	1 (0.1)	0 (0.0)	1 (0.2)	>0.999
Haemothorax, *n* (%)	5 (0.2)	3 (0.2)	2 (0.4)	0.297	3 (0.4)	1 (0.2)	2 (0.4)	>0.999
1 month	4 (0.2)	2 (0.1)	2 (0.4)	0.207	3 (0.4)	1 (0.2)	2 (0.4)	>0.999
1–12 months	1 (0.04)	0 (0.0)	1 (0.2)	0.217	1 (0.1)	0 (0.0)	1 (0.2)	>0.999
After 12 months	–	–	–	–	–	–	–	–
Pocket infection, *n* (%)	56 (2.2)	36 (1.8)	20 (3.7)	0.014	27 (2.5)	7 (1.3)	20 (3.7)	0.017
1 month	1 (0.04)	1 (0.1)	0 (0.0)	>0.999	0 (0.0)	0 (0.0)	0 (0.0)	>0.999
1–12 months	21 (0.8)	12 (0.6)	9 (1.6)	0.029	12 (1.1)	3 (0.5)	9 (1.6)	0.144
After 12 months	34 (1.4)	23 (1.2)	11 (2.0)	0.142	15 (1.4)	4 (0.7)	11 (2.0)	0.116
Infective endocarditis, *n* (%)	11 (0.4)	7 (0.4)	4 (0.7)	0.267	6 (0.5)	2 (0.4)	4 (0.7)	0.687
1 month	1 (0.04)	1 (0.1)	0 (0.0)	>0.999	1 (0.1)	1 (0.2)	0 (0.0)	>0.999
1–12 months	5 (0.2)	2 (0.1)	3 (0.5)	0.071	3 (0.3)	0 (0.0)	3 (0.5)	0.249
After 12 months	5 (0.2)	4 (0.2)	1 (0.2)	>0.999	2 (0.2)	1 (0.2)	1 (0.2)	>0.999

Categorical variables were listed as *n* (%). Categorical variables were compared using *χ*^2^ or Fisher’s exact tests. *P*-values refer to differences between the *de novo* and the upgrade CRT groups.

PSM, propensity score matching.

When these complications were investigated after performing propensity score matching, lead dysfunction or fracture [17 (3.1%) vs. 4 (0.7%); *P* = 0.007], and pocket infection [20 (3.7%) vs. 7 (1.3%); *P* = 0.017] remained more frequent among upgrade patients. Bleeding or pocket haematoma [12 (2.2%) vs. 16 (0.8%); *P* = 0.010] were more common complications in upgrade patients compared with the *de novo* CRT group, however no difference was observed after propensity score matching [12 (2.2%) vs. 6 (1.1%); *P* = 0.234]. Only the rate of pneumothorax was higher in the *de novo* group [28 (1.4%) vs. 2 (0.4%); *P* = 0.045], but this difference could not be confirmed when propensity score-matched groups were compared [8 (1.5%) vs. 2 (0.4%); *P* = 0.108]. Pneumothorax occurred more frequently in the first month following the procedure in the *de novo* CRT group [28 (1.4%) vs. 2 (0.4%); *P* = 0.045] than in upgrade CRT patients. Compared with the *de novo* CRT group, the incidence of bleeding [10 (1.8%) vs. 14 (0.7%); *P* = 0.024] at 1 month, lead dysfunction [5 (0.9%) vs. 3 (0.2%); *P* = 0.015], phrenic nerve stimulation [6 (1.1%) vs. 6 (0.3%); *P* = 0.028], and pocket infection [9 (1.6%) vs. 12 (0.6%); *P* = 0.029] at 1–12 months after the intervention was higher in the upgrade CRT group. In addition, the incidence of lead dysfunction [11 (2.0%) vs. 13 (0.7%); *P* = 0.010] was higher in the upgrade group 1 year after CRT implantation compared with the *de novo* group, which was also confirmed in the propensity score-matched cohort [11 (2.0%) vs. 2 (0.4%); *P* = 0.022] (*Table 2* and Supplementary material online, *Figure S1A* or *B*).

## Discussion

### Main findings

In our single-centre, high-volume analysis, we found that patients following CRT upgrade had a 41% higher risk of all-cause mortality/HTX/LVAD implantation and 43% higher risk of death from any cause compared with *de novo* CRT patients. However, after adjusting for relevant covariates using multivariable Cox regression analysis or propensity score matching, these differences disappeared. Regarding peri- and post-procedural complications, CRT upgrade procedures were associated with a higher rate of adverse events (such as lead dysfunction, bleeding, and infections) compared with *de novo* implantations.

### Patients characteristics

Several studies have highlighted the differences in the clinical characteristics between *de novo* and upgrade patients.^5^^,^^8–13^ As confirmed in these studies and our cohort, the latter group tends to have more comorbidities such as AF, ischaemic aetiology, previous ventricular arrhythmias, and chronic kidney disease.^5^^,^^8–13^ Furthermore, CRT upgrade patients are older, and this age difference corresponds roughly to the 3–5 years between the implantation of the first device and the upgrade procedure.^12^^,^^14^ Consequently, these observations can be partly attributable to the different aetiology of heart failure. Moreover, not only the age but also the RVP rate and the incidence of AF matter in this group.^6^ As previously described in the MOde Selection Trial (MOST), there is a linear relationship between the effect of cumulative ventricular pacing and the risk of developing AF.^6^

The renal function is also influenced by age.^15^ Although in one of the largest multicentre registries higher percentage of CKD was observed in *de novo* than upgrade patients,^16^ we found CKD to be more frequent in the latter group. Nonetheless, we have to emphasize that their population’s baseline characteristics differed vastly from those reported previously in the literature.^5^^,^^8–10^ Accompanying the high prevalence of CKD, serum creatinine levels were significantly higher among upgrade CRT patients than the *de novo* group in our cohort. Parallel to our findings, Wokhlu *et al*.^8^ have also highlighted the importance of baseline serum creatinine levels as an independent predictor of mortality in a similar population.

Although women are underrepresented in CRT trials (as females account for ∼19–27% of the study populations), they show a better response to CRT than men.^5^^,^^11^ In our total cohort, there were 25% female candidates. The rate of female sex was lower in the upgrade than the *de novo* group (20% vs. 27%; *P* = 0.002).

### Differences in long-term outcome

Data regarding the differences in long-term mortality between *de novo* and upgrade CRT patients are scarce. Smaller, short-term observational studies have failed to demonstrate significant differences in survival,^8^^,^^14^ and the analyses of larger registries did not provide consistent results.^5^^,^^13^^,^^14^ In one of the largest observational registries comparing 692 upgrade and 1675 *de novo* patients, no significant difference was found in the total and cause-specific mortality after 1-year follow-up time.^13^ However, in another multicentre, observational, prospective study, opposite results were reported, with more beneficial outcomes among *de novo* CRT patients, which persisted even after propensity score matching.^12^ Nevertheless, in this study, only CRT-D recipients were included, and as these patients are generally younger and less vulnerable, this fact may also affect the mid-term outcomes.

Leyva *et al*. included CRT patients from 2000 to 2016, and they found a significantly higher rate of all-cause mortality and mortality/HF hospitalization in upgrade patients. However, after performing multivariable Cox proportional hazard analysis or propensity score matching, no differences could be observed in these endpoints.^10^ Their univariable analysis proved that all-cause mortality was higher in upgrade than *de novo* patients in men and subgroups of patients with NYHA III functional class, CRT-P, non-ischaemic cardiomyopathy, non-diabetic status, LBBB, QRS ≥150ms, and LVEF ≤25%. These results are in line with ours, as we also found a higher risk of the composite endpoint in these subgroups among upgrade CRT patients. Furthermore, in the subgroups of age between 60 and 74 years, female, NYHA functional Class I–II, CRT-D, ischaemic heart failure, with or without chronic kidney disease, AF, and LVEF >28%, the risk of reaching the composite endpoint was higher in upgrade patients than the *de novo* groups. After comparing the propensity score-matched cohorts, we found less favourable outcomes among upgrade patients in the NYHA III–IVa functional class subgroup than in the *de novo* group. Moreover, Leyva *et al*. observed a higher risk of mortality in upgrade patients receiving CRT-P devices than those being upgraded to a CRT-D.^10^ Compared with our patient cohort, Leyva *et al*. included CRT upgrade recipients with no history of sustained ventricular arrhythmia, and they investigated the CRT upgrade in the context of primary prevention. In contrast, we included patients both with and without prior ventricular arrhythmia.

Notably, not only the type of the implanted device but also the date of implantations might affect the outcomes due to the continuously improving device technology and drug treatment. However, neither the previously reported data^10^ nor our results could confirm that the date of implantation has an impact on outcomes.

Previously, we conducted a systematic review and meta-analysis comprising 468 205 *de novo* and 21 363 upgrade CRT patients, in which we observed a similar risk of all-cause mortality in these two patient groups (risk ratio 1.19; 95% CI: 0.88–1.60; *P* = 0.27).^14^ This finding was confirmed by the current analysis after adjusting for relevant covariates or performing propensity score matching.

### Peri-procedural and long-term complications

Although data on the comparison of complications associated with *de novo* and upgrade CRT procedures are scarce,^5^^,^^13^^,^^14^ CRT upgrade is considered to be a procedure with a higher complication rate. Our analysis showed higher rates of lead dysfunction, bleeding, haematoma, or pocket infection among upgrade patients, even after propensity score matching. Similar to our findings, in the REPLACE registry and the RAFT Upgrade Substudy, the most common complication was lead displacement or dysfunction in both CRT groups.^17^^,^^18^ During upgrade procedures, the risk of damaging the previously implanted leads or having any difficulties with the newly implanted ones is higher than in *de novo* patients.^19^ This may explain our observation that compared with *de novo*, upgrade CRT procedures were associated a higher prevalence of lead dysfunction [11 (2.0%) vs. 2 (0.4%); *P* = 0.022] after 1 year.

Cardiac resynchronization therapy implantation, especially in older age or in patients with coagulopathy (renal insufficiency, anticoagulant intake), is associated with an increased risk of developing a post-procedural pocket haematoma^20^ and subsequent infections, as proved in our study cohort as well.

In the current analysis, only pneumothorax (PTX) could be observed more frequently (1.4% vs. 0.4%; *P* = 0.045) in *de novo* patients, but this difference vanished after propensity score matching. This phenomenon can be explained by the presence of the previously implanted leads, which may help identify the subclavian vein.

However, neither the largest observational registries, the European CRT Survey,^13^ and the European CRT Survey II^11^ nor other small observational studies found any significant differences between the two patient groups in terms of complications.^12^^,^^14^ The inconsistency of data implies that not only the characteristics of the patient cohort but also the experience of the implanting physicians and the duration of procedure should be taken into account.^19^

### Limitations

Our study has certain limitations. First, we performed our analyses in a retrospective manner. Thus, the groups were unbalanced in some aspects. To adjust for these differences, we performed multivariable Cox regression analysis and propensity score matching besides the univariable analyses. Moreover, due to the retrospective nature of the study, data were missing in a moderate proportion of patients. Secondly, as the study covers >19 years, general therapeutic protocols, lead choice, device programming, medical treatment options, technical equipment, and guidelines have changed over time. However, when we investigated the impact of the implantation date on outcomes, no significant effect could be observed.

## Conclusions

Patients who underwent upgrade CRT exhibited worse outcomes compared with the *de novo* implantation group. Nevertheless, this difference may be attributable to the higher comorbidity burden of upgrade patients. The rate of peri- and post-procedural complications (i.e. lead dysfunction and pocket infections) was higher among upgrade patients.

## Supplementary material

Supplementary material is available at *Europace* online.

## Supplementary Material

euab059_Supplementary_Data
