# Supplementary material for: Long-term survival following upgrade compared with de novo cardiac resynchronization therapy implantation: a single-centre, high-volume experience
Source: Europace. 2021 May 25;23(8):1310–8. doi: 10.1093/europace/euab059 (PMC8350864; doi:10.1093/europace/euab059)

***SUPPLEMENTAL MATERIAL***

**Supplemental Table S1*.*** Multivariable Cox regression analysis: predictors of the primary composite endpoint in de novo vs. upgrade CRT patient groups (ACE-I or ARB, atrial fibrillation, amiodarone, De novo vs. upgrade, female vs. male sex, ischemic vs. non-ischemic heart failure etiology, LVEDd, LVESd, LVEF, NYHA class I-II. Vs. III-IV., OAC, QRS duration time, Serum Creatinine, Ventricular arrhythmia)

|  | **Multivariable HR (95% CI)** | **P-value** |
| --- | --- | --- |
| ACE-I or ARB | 0.76 (0.54-1.07) | 0.110 |
| Age | 1.01 (1.00-1.02) | 0.052 |
| Atrial fibrillation | 1.31 (1.02-1.69) | 0.032 |
| Amiodarone | 1.14 (0.89-1.47) | 0.304 |
| Upgrade | 1.12 (0.86-1.48) | 0.402 |
| Female sex | 0.72 (0.54-0.96) | 0.025 |
| Ischemic heart failure etiology | 1.66 (1.32-2.09) | <0.001 |
| LVEDd | 0.98 (0.95-1.02) | 0.281 |
| LVESd | 1.04 (1.00-1.07) | 0.051 |
| LVEF | 0.99 (0.98-1.02) | 0.689 |
| NYHA class III-IV. | 1.38 (1.09-1.75) | 0.009 |
| OAC | 1.03 (0.80-1.32) | 0.825 |
| QRS duration time | 0.99 (0.99-1.00) | 0.434 |
| Serum Creatinine | 1.01 (1.01-1.00) | <0.001 |
| Ventricular arrhythmia | 0.87 (0.68-1.12) | 0.278 |

ACE-I, angiotensin-converting-enzyme inhibitors; ARB, angiotensin receptor blocker; CI, Confidence interval; HR, Hazard ratio; LVEDd, left ventricular end-diastolic diameter; LVEF, left ventricular ejection fraction; LVESd, left ventricular end-systolic diameter; NYHA, New York Heart Association class; OAC, oral anticoagulant

**Supplemental Table S2*.*** Baseline characteristics after propensity score matching

|  | De novo CRT (n=547) | Upgrade CRT (n=547) | P-value |
| --- | --- | --- | --- |
| Age (year; mean ± SD) | 70.2 ± 9.4 | 70.6 ± 9.2 | 0.528 |
| Atrial fibrillation (n; %) | 255 (47%) | 258 (47%) | 0.904 |
| eGFR (mL/min/1.73m^2^; mean ± SD) | 56.7 ± 21 | 55.7 ± 21.5 | 0.321 |
| HF etiology (n; %) | 307 (56%) | 328 (60%) | 0.220 |
| LVEF (%; mean ± SD) | 29.3 ± 7.0 | 29.4 ± 7.9 | 0.885 |
| NYHA II (n; %) | 257 (50%) | 247 (48%) | 0.313 |
| NYHA III (n; %) | 229 (45%) | 231 (45%) |  |
| NYHA IV (n; %) | 23 (5%) | 34 (7%) |  |
| Male (n; %) | 425 (78%) | 437 (80%) | 0.416 |
| QRS duration time (ms; mean ± SD) | 173.7 ± 27.8 | 174.1 ± 30.6 | 0.821 |
| Ventricular arrhythmia (n; %) | 193 (35%) | 181 (33%) | 0.511 |

Continuous variables were listed as mean ± SD, and categorical variables were listed as n (%). Continuous variables were compared using unpaired Student’s *t*-test, while categorical variables were compared using Chi-squared or Fisher’s exact tests. P-values refer to differences between the de novo and the upgrade CRT groups.

eGFR, estimated glomerular filtration rate; HF, heart failure; LVEF, left ventricular ejection fraction; NYHA, New York Heart Association class; SD, standard deviation

**Supplemental Table S3*.*** Multivariable Cox regression analysis: predictors of all-cause mortality in de novo vs. upgrade CRT patient groups (ACE-I or ARB, atrial fibrillation, amiodarone, De novo vs. upgrade, female vs. male sex, ischemic vs. non-ischemic heart failure etiology, LVEDd, LVESd, LVEF, NYHA class I-II. Vs. III-IV., OAC, QRS duration time, Serum Creatinine, Ventricular arrhythmia)

|  | **Multivariable HR (95% CI)** | **P-value** |
| --- | --- | --- |
| ACE-I or ARB | 0.74 (0.74 – 1.04) | 0.081 |
| Age | 1.02 (1.01 – 1.03) | 0.002 |
| Atrial fibrillation | 1.41 (1.10 – 1.81) | 0.008 |
| Amiodarone | 1.13 (0.88 – 1.46) | 0.344 |
| Upgrade | 1.10 (0.84 – 1.45) | 0.489 |
| Female sex | 0.74 (0.56 – 0.99) | 0.042 |
| Ischemic heart failure etiology | 1.59 (1.26 – 2.00) | <0.001 |
| LVEDd | 0.98 (0.95 – 1.02) | 0.271 |
| LVESd | 1.04 (1.00 – 1.08) | 0.039 |
| LVEF | 0.99 (0.98 – 1.02) | 0.703 |
| NYHA class III-IV. | 1.35 (1.06 – 1.71) | 0.015 |
| OAC | 0.99 (0.77 – 1.27) | 0.950 |
| QRS duration time | 0.99 (0.99 – 1.00) | 0.675 |
| Serum Creatinine | 1.01 (1.00 – 1.01) | <0.001 |
| Ventricular arrhythmia | 0.88 (0.68 – 1.13) | 0.302 |

ACE-I, angiotensin-converting-enzyme inhibitors; ARB, angiotensin receptor blocker; CI, Confidence interval; HR, Hazard ratio; LVEDd, left ventricular end-diastolic diameter; LVEF, left ventricular ejection fraction; LVESd, left ventricular end-systolic diameter; NYHA, New York Heart Association class; OAC, oral anticoagulant

**Supplemental Figure 1A.** The occurrence of complications in the de novo CRT group during follow-up

**Supplemental Figure 1B.** The occurrence of complications in the upgrade CRT group during follow-up

The dashed lines indicate the first and twelfth months of the follow-up period. Each given point indicates the occurrence of a complication associated with (A) de novo and (B) upgrade CRT implantation.


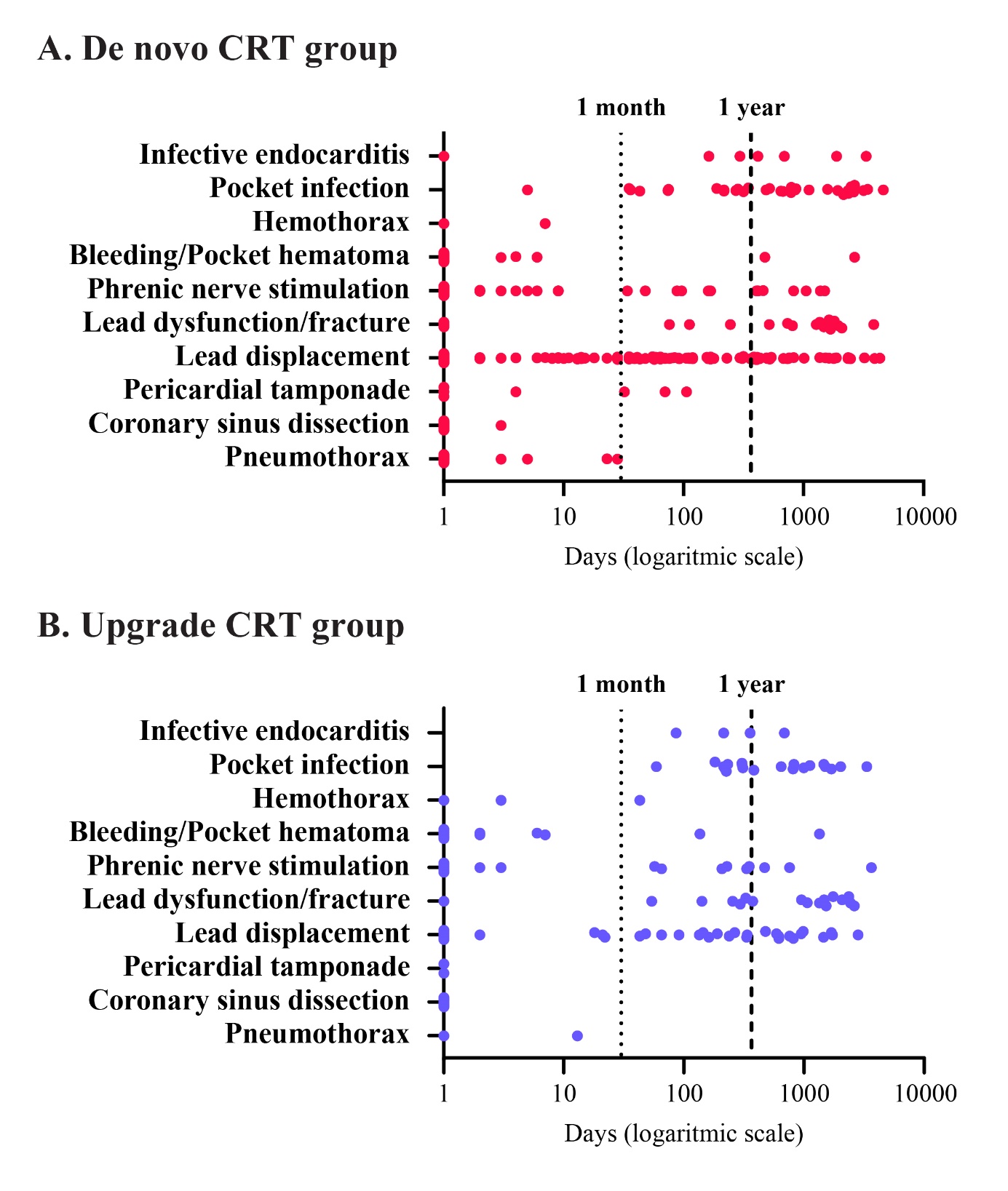

Supplement: euab059_Supplementary_Data [file euab059_supplementary_data.zip › 30.01. Supplementary file Clean version.docx]
