# Supplementary figures and images for: Long-term survival following upgrade compared with de novo cardiac resynchronization therapy implantation: a single-centre, high-volume experience
Source: Europace. 2021 May 25;23(8):1310–8. doi: 10.1093/europace/euab059 (PMC8350864; doi:10.1093/europace/euab059)

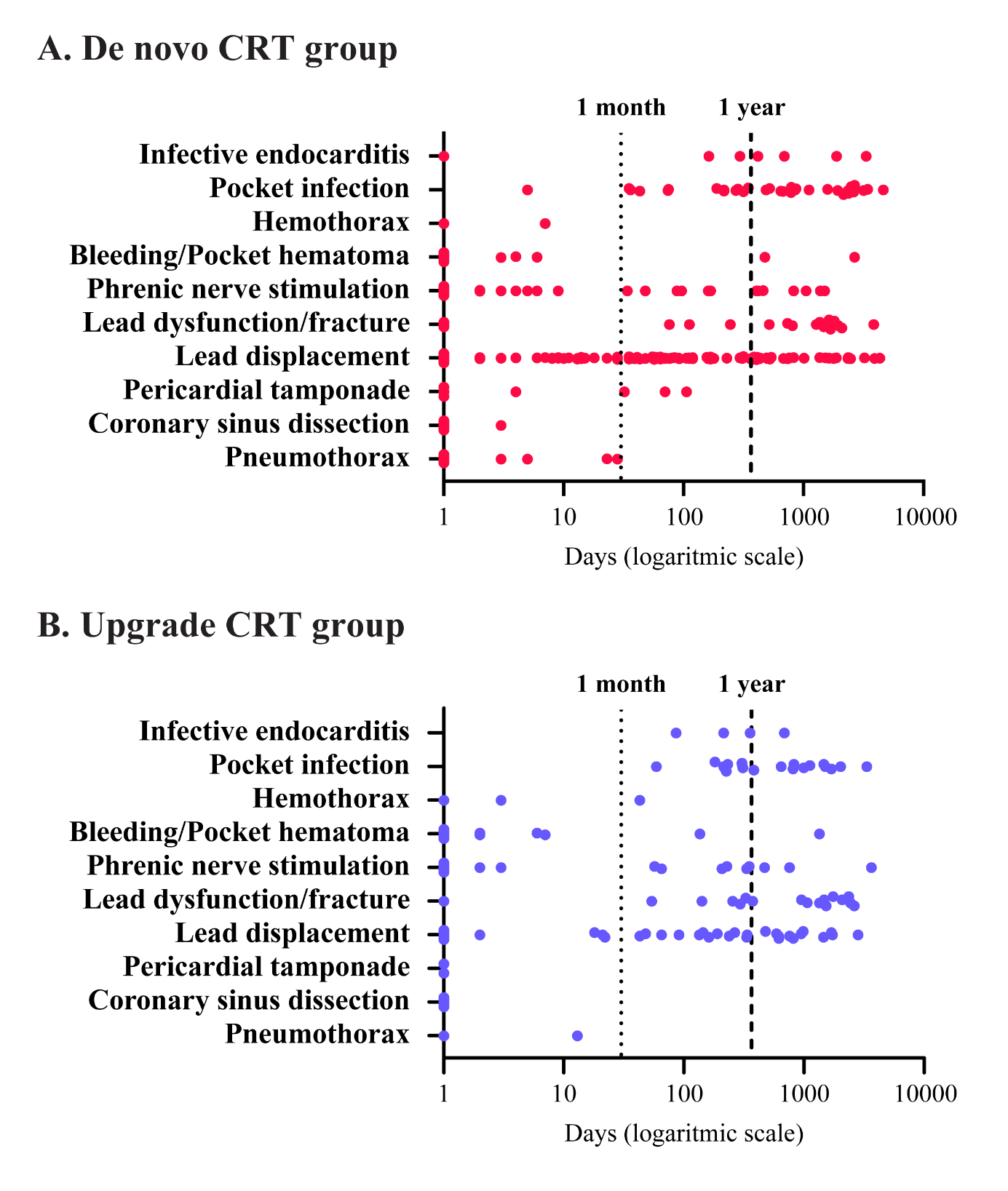

Supplement: euab059_Supplementary_Data [file euab059_supplementary_data.zip › Supplementary Figure 1.tif]
